# ANP promotes proliferation and inhibits apoptosis of ovarian granulosa cells by NPRA/PGRMC1/EGFR complex and improves ovary functions of PCOS rats

**DOI:** 10.1038/cddis.2017.494

**Published:** 2017-10-26

**Authors:** Qin Zheng, Yulin Li, Dandan Zhang, Xinyuan Cui, Kuixing Dai, Yu Yang, Shuai Liu, Jichun Tan, Qiu Yan

**Affiliations:** 1 Department of Biochemistry and Molecular Biology, Dalian Medical University, Liaoning Provincial Core Lab of Glycobiology and Glycoengineering, Dalian 116044, China; 2 Centre for Auxiliary Human Reproduction, Shengjing Hospital of China Medical University, Shenyang 110004, China

## Abstract

Polycystic ovary syndrome (PCOS) is a complicated reproductive endocrine disease characterized by polycystic ovaries, hyperandrogenism and anovulation. It is one of the main causes of infertility. RU486 is an antagonist of progesterone receptor, and most commonly used as a contraceptive. However, whether RU486 is correlated with PCOS remains unclear. Atrial natriuretic peptide (ANP) is a small peptide with natriuretic and diuretic functions, and its availability to be used in PCOS treatment is unknown. Here, we showed that the serum ANP level was lower in PCOS patients than that in healthy women, and it was also decreased in the serum and ovarian tissues of RU486-induced PCOS rats compared with the control rats. We also found that RU486 inhibited the proliferation and promoted the apoptosis of human KGN ovarian granulosa cells by downregulating progesterone receptor membrane component 1 (PGRMC1). Meantime, ANP promoted the proliferation and inhibited the apoptosis of KGN cells through upregulating ANP receptor A (NPRA). The promotive effects of ANP on ovarian functions were mediated through the formation of an NPRA/PGRMC1/EGFR complex, which further activated MAPK/ERK signaling and transcription factor AP1. Moreover, ANP treatment reversed the PCOS symptoms, and improved the fertility of RU486-induced PCOS rats. Collectively, these findings highlight that RU486 is associated with the pathogenesis of PCOS, and ANP treatment may be a promising therapeutic option for PCOS.

Polycystic ovary syndrome (PCOS) is one of the most incident reproductive endocrine diseases, with a prevalence ranging from 5 to 10% in women of reproductive age.^1^ The typical characteristics of PCOS include polycystic ovaries, hyperandrogenism and anovulation.^2^ Although the pathogenesis of PCOS is complex and largely unknown, the syndromes are often associated with hormone disorders, such as decreased progesterone and increased testosterone, estrogen and luteinizing hormone (LH), etc.^3^ Progesterone is a steroid hormone secreted mainly by ovarian granulosa cells and luteal cells. The function of progesterone is associated with follicular maturation,^4^ ovulation,^5^ embryonic development,^6^ endometrial receptivity and embryo implantation.^7^ It is also considered as an essential hormone for pregnancy maintenance.^8^ Patients with PCOS fail to form a corpus luteum, leading to a low level of progesterone and infertility.^9, 10^ Progesterone dysfunction likely plays an important role in the pathophysiology of PCOS.

RU486 (mifepristone), a progesterone receptor antagonist, is an effective and the most commonly used contraceptive. The structure of RU486 is similar to that of progesterone, but its binding affinity to progesterone receptor is five times stronger than that of progesterone to progesterone receptor.^11^ Therefore, RU486 can strongly block the progesterone functions by competitively binding to progesterone receptor. Furthermore, RU486 inhibits the development and maturation of follicles, resulting in the delayed occurrence of ovulation.^12^ Brown *et al.*^13^ found that most of the women exhibited anovulation after the continuous administration of RU486 (2  or 5 mg/day) for 120 days, indicating that RU486 inhibited the development of follicles and ovulation. Other studies reported that the use of RU486 impaired corpus luteum formation and decreased progesterone production.^14^ Moreover, the development of the uterine endometrium and the establishment of endometrial receptivity, as well as the rate of pregnancy could also be influenced by RU486.^15^ Therefore, we speculated that the abuse of RU486 might be an etiological factor of PCOS, which was also correlated with infertility.

Recently, a new type of progesterone receptor was discovered: progesterone receptor membrane component (PGRMC), including PGRMC1 and PGRMC2.^16^ The expression of PGRMC1 is higher than that of PGRMC2, and it is mainly found in ovarian granulosa cells and luteal cells.^17^ PGRMC1 is also present in the fallopian tubes,^18^ endometrium^19^ and placenta.^20^ Knockdown of PGRMC1 expression in mouse ovarian granulosa cells significantly reduced the number of antral follicles in the ovaries and the antiapoptotic capacity of progesterone, indicating that PGRMC1 may be involved in the growth and development of the ovaries,^21^ and the regulation of progesterone functions for granulosa cells.^22^ Abnormal PGRMC1 expression causes diseases, such as cancer^23^ and infertility,^24^ etc. Schuster *et al.*^25^ compared the peripheral blood level of PGRMC1 level in women with regular menstrual cycle, PCOS and premature ovarian failure (POF) patients, and showed that the PGRMC1 level was lower in the PCOS and POF patients than that in the women with regular menstrual cycle.^25^ The results reveal that PGRMC1 may be associated with PCOS.

Atrial natriuretic peptide (ANP), a member of the natriuretic peptide family, is mainly secreted by atrial myocytes, which plays essential roles in regulating blood pressure, the salt and water balance and body fluid homeostasis based on its pharmacological functions in natriuresis and diuresis.^26^ In addition to atrial myocytes, ANP has also been found in ovarian oocytes,^27^ granulosa cells^28^ and the corpus luteum.^29^ Ovary-derived ANP can regulate ovarian functions, such as follicular growth and hormone production, through autocrine or paracrine manner.^30^ The serum ANP level was decreased in PCOS patients.^31^ Moreover, PCOS showed ovarian polycystic and physalides phenotypes. Taken together, these led us to hypothesize that ANP may be a novel therapeutic strategy for PCOS patients. The diverse physiological functions of ANP are manifested by its binding to the specific cell surface receptors. There are ANP-specific binding sites on ovarian granulosa cells and luteal cells.^32, 33^ The binding of ANP to its specific receptors activates the downstream signaling pathways which are related to cell growth and apoptosis, etc. To date, three different subtypes of natriuretic peptide receptors (NPRs) have been identified: NPRA, NPRB and NPRC.^34^ Among the different NPRs, ANP shows the highest affinity for NPRA. ANP and NPRA were involved in the fertilization process. Hotchkiss *et al.*^35^ found that NPRA was expressed on blastocysts and embryonic stem cells, and involved in maintaining embryonic stem cells self-renewal and pluripotency. Zhang *et al.*^36^ also reported that the binding of ANP and NPRA induced sperm acrosome reaction occurred, whereas the receptor antagonist could eliminate the effect of ANP.

Till now, there is still a lack of effective therapies with low side effects because clinical presentations are complex and variable among PCOS patients.^37^ The major clinical treatments of PCOS patients include ovulation induction, anti-androgen, metabolic abnormality correction and ovarian drilling operation, etc. The main drugs used for PCOS is steroid hormones, which have many side effects. Hence, it is necessary to explore the etiology of PCOS and search for new non-steroid hormonal replacements to provide ideal treatment options for PCOS patients. ANP is a 28-amino-acid polypeptide, and its application in reproductive diseases has not yet been investigated. In the current study, we found that the serum ANP level was lower in PCOS patients than that in healthy women, and it was also reduced in the serum and ovary tissues of RU486-induced PCOS rats compared with that in control rats. ANP promoted the expression of NPRA and the formation of NPRA/PGRMC1/EGFR complex. Moreover, ANP treatment not only improved the morphology and functions of the ovary but also ameliorated the receptivity of the uterine endometrium and the pregnancy rate of PCOS rats.

## Results

### Decreased level of ANP in PCOS patients and PCOS rats, and improvement of ovary morphology and functions after ANP treatment

**Figure 1:**
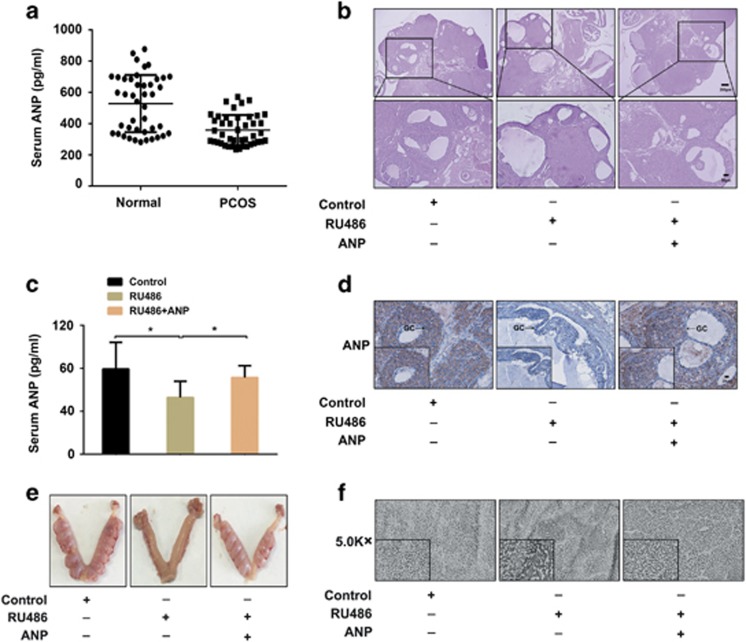
Expression of ANP in serum of PCOS patients and RU486-induced PCOS rats. (**a**) Analysis of ANP level in serum of PCOS patients and healthy women by ELISA. (**b**) Representative HE staining of the ovaries from RU486-induced PCOS rats and ANP treatment group. Bar=200 *μ*m or Bar=50 *μ*m. (**c**) Analysis of ANP in serum of PCOS rats and ANP treatment group. (**d**) Immunohistochemical staining of ANP in PCOS rats and ANP treatment group. Bar=20 *μ*m. Significance was indicated by *P<0.05. (**e**) Implanted embryos in PCOS rats and ANP-treated rats. (**f**) Microvilli on uterine endometrium detected by scanning electron microscopy (SEM) in differently treated groups

We first compared the level of ANP in the serum of PCOS patients and healthy women by ELISA (Figure 1a). The results showed that the serum ANP level in PCOS patients was significantly lower than that in healthy women. To confirm the alteration of ANP was associated with PCOS phenotypes, the RU486-induced PCOS rat model was used. As shown in Figure 1b, RU486-treated rats presented PCOS phenotypes, including polycystic, enlarged ovaries and thinner granular cell layer, etc. The changed serum hormone levels (Testosterone(T), Progesterone (P) and Estradiol (E_2_)) also testified the pathogenesis of PCOS rats (Table 1). We further found that serum and ovarian ANP was significantly decreased in PCOS rats, compared with the control (Figures 1c and d), which was consistent with that of PCOS patients. Therefore, we hypothesized that ANP administration in PCOS rats may improve the ovarian morphology and functions. In the ANP treatment group, the thicker granular cell layer and elevated ANP expression were observed by immunohistochemical staining (Figure 1d). Additionally, to explore whether the embryo implantation potential was affected by the ANP level, we detected the alterations in implanted embryo number at gestational day (GD) 10 and ultrastructural changes in the uterine endometrium at GD4 by SEM. The results showed that the number of implanted embryos in the RU486 group was notably reduced, compared with that in the ANP treatment group (Figure 1e). The microvilli on the endometrial surface were decreased in the RU486 group, but recovered in the ANP group (Figure 1f). The above data suggest that ANP can be used as a new therapeutic drug for PCOS.

### Co-expression of NPRA/NPRC and PGRMC1 in human ovarian granulosa cells and ovary tissues of PCOS rats

**Figure 2:**
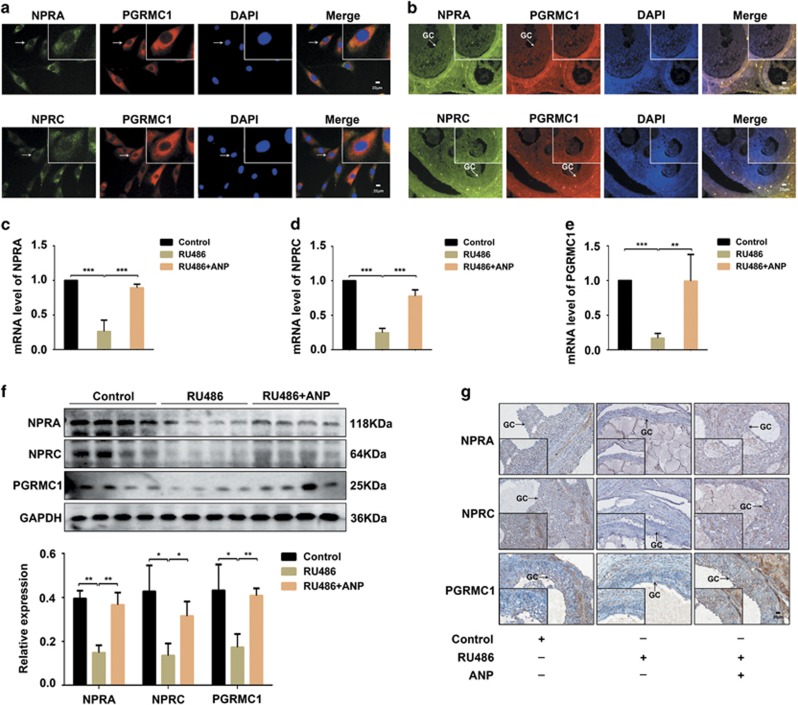
Co-expression of NPRA/NPRC and PGRMC1 in ovarian granulosa cells and PCOS rat model. (**a**) Representative immunofluorescent staining of NPRA/NPRC and PGRMC1 in human ovarian granulosa cells (KGN). NPRA and NPRC were visualized as green, and PGRMC1 as red. The nuclei were stained with DAPI (blue). Bar=20 *μ*m. (**b**) Representative images of Immunofluorescent staining of NPRA/NPRC and PGRMC1 in ovarian granulosa cells in rat. Green: NPRA/NPRC; Red: PGRMC1; Blue: DAPI. Bar=20 *μ*m. (**c, d** and **e**) mRNA levels of NPRA, NPRC and PGRMC1 in the ovary tissues of RU486-induced PCOS rats and the ANP treatment group by real-time PCR. (**f**) Expression and analysis of NPRA, NPRC and PGRMC1 in the ovary tissues of RU486-induced PCOS rats and the ANP treatment group by western blot. (**g**) Representative images of immunohistochemical staining of NPRA, NPRC and PGRMC1 in ovarian granulosa cells of PCOS and ANP-treated rats. Bar=50 *μ*m. Significance was indicated by *P<0.05, **P<0.01, ***P<0.001

Based on the above results of decreased levels of ANP and progesterone in PCOS patients and rat model, we further detected the location and expression of NPRA/C and PGRMC1 in human ovarian granulosa cells (KGN) and ovary tissues of rats. The analysis of immunofluorescent staining showed that NPRA and PGRMC1, or NPRC and PGRMC1 were co-localized both in KGN cells (Figure 2a) and ovary tissues of rats (Figure 2b). The expression of NPRA, NPRC and PGRMC1 was lower in the ovary of PCOS rats than the control, whereas the levels were reversed in the ANP treatment group by real-time PCR and western blot (Figures 2c–f). Similar results were found in the expression changes of NPRA, NPRC and PGRMC1 in ovary tissues of rats of PCOS and the ANP group by immunohistochemical staining (Figure 2g). Our findings indicate that co-expression of NPRA/C and PGRMC1 may be involved in regulating ovary functions.

### Downregulating the expression of PGRMC1 by RU486 inhibited the proliferation and promoted the apoptosis of human ovarian granulosa cells

**Figure 3:**
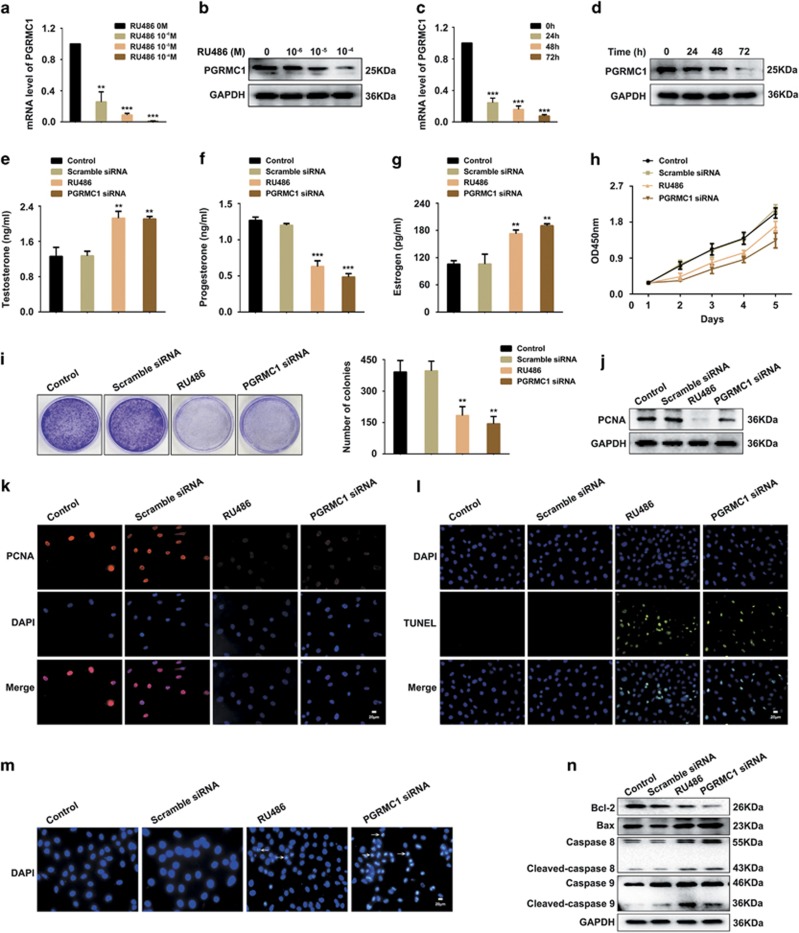
Downregulated PGRMC1 by RU486 suppressed proliferation and promoted apoptosis of KGN cells. (**a** and **b**) KGN cells were treated with different concentrations of RU486 (0, 10^−6^, 10^−5^, 10^−4^ M) for 48 h. mRNA and protein levels of PGRMC1 were detected by real-time PCR and western blot. (**c** and **d**) KGN cells were treated with RU486 at the indicated times (0, 24, 48, 72 h). Expression of PGRMC1 in KGN cells was analyzed by real-time PCR and western blot. (**e-g**) KGN cells were treated with RU486, transfected with scramble siRNA or PGRMC1 siRNA, respectively. Analysis of testosterone, progesterone and estrogen in the culture medium by ELISA. (**h**) CCK-8 assay for cell proliferation. (**i**) Colony formation assay. Representative images and statistical analysis were showed. (**j**) Protein level of PCNA in KGN cells was detected by western blot. (**k**) Immunofluorescent staining of PCNA in KGN cells. Red: PCNA; blue: DAPI. Bar=20 *μ*m. (**l**) Apoptosis analysis by TUNEL staining in KGN cells. Blue: DAPI; green: TUNEL. Bar=20 *μ*m. (**m**) DAPI staining for apoptosis analysis of KGN cells. Bar=20 *μ*m. (**n**) Western blot analysis of apoptosis-related proteins in KGN cells. Significance was indicated by **P<0.01, ***P<0.001

To investigate the correlation and the roles of RU486 and PGRMC1 in ovary functions, KGN cells were treated with different RU486 concentrations (0, 10^−6^, 10^−5^, 10^−4^ M) for different times (0, 24, 48, 72 h). The results showed that RU486 significantly decreased the mRNA and protein levels of PGRMC1 in KGN cells (Figures 3a–d). The culture medium of KGN cells treated with RU486, transfected with scramble RNA or PGRMC1 siRNA were collected to determine the alterations of hormone secretion. The ELISA results showed that both RU486 and PGRMC1 siRNA increased the secretion of testosterone (Figure 3e) and estrogen (Figure 3g), but decreased the level of progesterone (Figure 3f). Then, we estimated the effects of RU486 and PCRMC1 on cell proliferation using CCK-8 (Figure 3h) and colony formation assay (Figure 3i). Compared with the control group, RU486 significantly weakened the proliferation ability of KGN cells, as did by PGRMC1 siRNA transfection. PCNA expression was downregulated after RU486 addition or PGRMC1 siRNA transfection as detected by western blot (Figure 3j) and immunofluorescent staining (Figure 3k). The apoptosis of KGN cells was analyzed by TUNEL staining (Figure 3l), DAPI staining (Figure 3m) and western blot (Figure 3n). Both RU486 and PGRMC1 siRNA treatments resulted in much higher rates of apoptosis than that of the control group. The treatments also downregulated the expression of the antiapoptosis protein Bcl-2, while upregulating the expression of the pro-apoptosis proteins Bax, caspase 8 and caspase 9. The above data reveal that both RU486 and decreased expression of PGRMC1 can impair the functions of ovary cells.

### ANP upregulated NPRA/C expression and promoted proliferation and inhibited apoptosis of KGN cells

**Figure 4:**
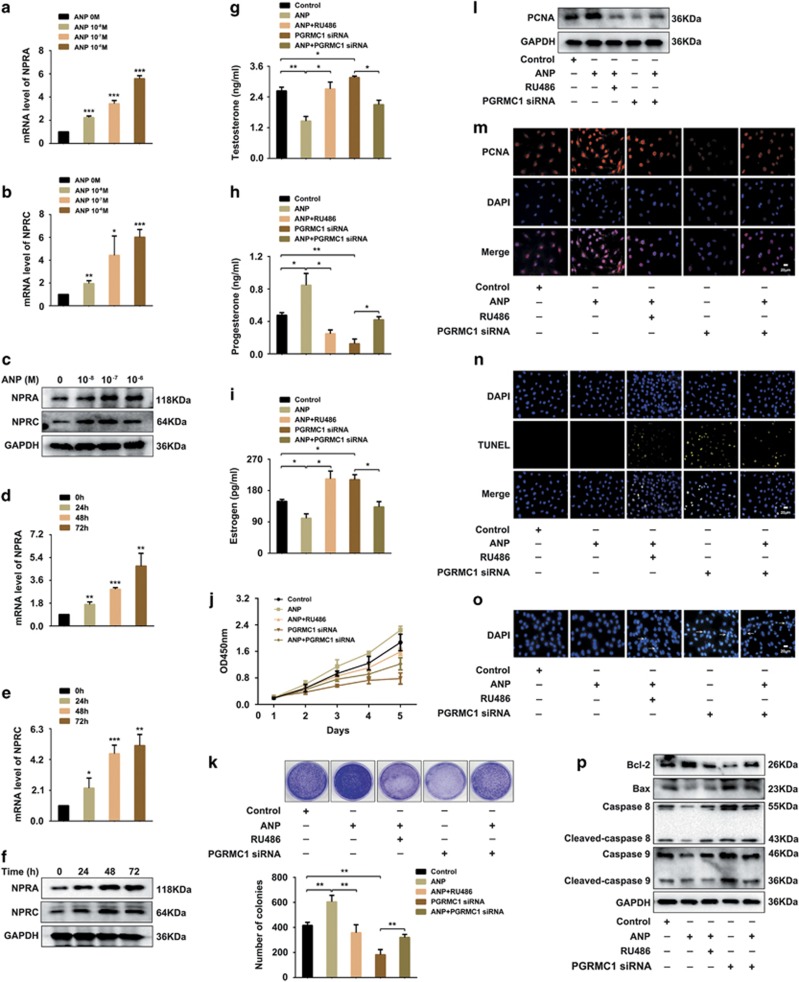
ANP upregulated NARA/C expression and promoted the proliferation and inhibited the apoptosis of KGN cells. (**a-c**) KGN cells were treated with different concentrations of ANP (0, 10^−8^, 10^−7^, 10^−6^ M) for 48 h. mRNA and protein levels of NPRA/C were detected by real-time PCR and western blot. (**d–f**) KGN cells were treated with ANP at the indicated times (0, 24, 48, 72 h). mRNA and protein levels of NPRA/C detected by real-time PCR and western blot. (**g–i**) KGN cells were treated with ANP, or ANP and RU486, PGRMC1 siRNA, or ANP and PGRMC1 siRNA, respectively. Levels of testosterone, progesterone and estrogen were measured by ELISA in the culture medium of KGN cells. (**j**) CCK-8 assay for cell proliferation. (**k**) Colony formation assay. Representative images and statistical analysis were showed. (**l**) Western blot analysis of protein level of PCNA in KGN cells. (**m**) Immunofluorescent staining of PCNA in KGN cells. Red: PCNA; Blue: DAPI. Bar=20 *μ*m. (**n**) TUNEL staining for apoptosis analysis of KGN cells. Blue: DAPI; Green: TUNEL. Bar=20 *μ*m. (**o**) DAPI staining for morphological evaluation of apoptotic cells. Bar=20 *μ*m. (**p**) Western blot analysis of apoptosis-related proteins in KGN cells. Significance was indicated by *P<0.05, **P<0.01, ***P<0.001

We then investigated whether ANP could affect ovarian functions by regulating the expression of NPRA/C in KGN cells. The results revealed that ANP increased the expression levels of NPRA (Figures 4a, c, d and f) and NPRC (Figures 4b, c, e and f) both in mRNA and protein levels. The culture medium of KGN cells treated with ANP, RU486 or PGRMC1 siRNA was collected for the hormone analysis. ELISA results showed that the ANP treatment decreased the secretion of testosterone (Figure 4g) and estrogen (Figure 4i), but increased the level of progesterone (Figure 4h), and partly restored the alterations in three kinds of hormones caused by RU486 and PGRMC1 siRNA treatment. The effect of ANP on cell proliferation was detected by CCK-8 and colony formation assay. These results indicated that ANP accelerated the growth of KGN cells, and partially recovered the inhibitory effect by RU486 or PGRMC1 siRNA (Figures 4j and k). The analysis of PCNA expression by western blot (Figure 4l) and immunofluorescent staining (Figure 4m) further confirmed the effect of ANP promoting cell proliferation potential. TUNEL staining (Figure 4n), DAPI staining (Figure 4o) and western blot (Figure 4p) were applied to determine the influence of ANP on cell apoptosis. The results showed that ANP upregulated Bcl-2 expression and downregulated Bax, caspase 8 and caspase 9 expression, while RU486 and PGRMC1 siRNA had opposite effects. The results suggest that ANP could facilitate proliferation and reduce the apoptosis of ovarian granulosa cells by upregulation of NPRA/C expression.

### ANP promoted the proliferation of human ovarian granulosa cells through NPRA/PGRMC1/EGFR complex formation and activating MAPK/ERK signaling pathway

**Figure 5:**
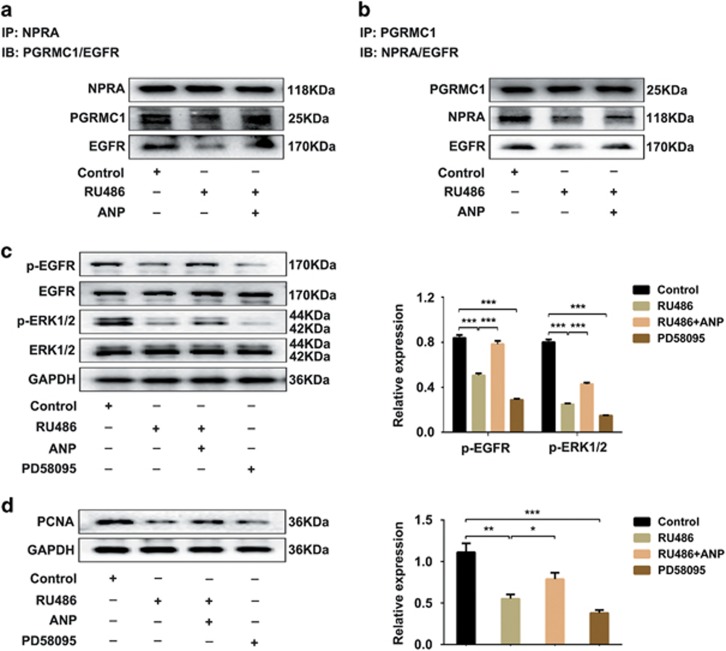
NPRA/PGRMC1/EGFR complex promoted the proliferation of ovarian granulosa cells through the MAPK/ERK signaling pathway. KGN cells were treated with RU486, or RU486 and ANP, respectively. (**a**) Immunoprecipitation (IP): anti-NPRA pulled down proteins. Immune blot (IB): levels of PGRMC1 and EGFR were detected by western blot. (**b**) IP: anti-PGRMC1 pulled down proteins. IB: levels of NPRA and EGFR were detected by western blot. (**c**) KGN cells were treated with RU486, RU486 and ANP, or the inhibitor of MAPK/ERK (PD58095). Phosphorylation of EGFR (p-EGFR) and ERK1/2 (p-ERK1/2) were detected by western blot. (**d**) Western blot analysis of PCNA expression in KGN cells. Statistical analysis was shown. Significance was indicated by *P<0.05, **P<0.01, ***P<0.001

To explore the molecular mechanism underlying the ANP-mediated regulation of ovarian functions, the interactions among NPRA, PGRMC1 and EGFR, as well as the activation of MAPK/ERK signaling pathway were analyzed. KGN cells were pretreated with RU486 with or without ANP, and then immunoprecipitation (IP) was used to detect the relationships among NPRA, PGRMC1 and EGFR. As shown in Figures 5a and b, NPRA, PGRMC1 and EGFR could form a binding complex. It was further confirmed by the regulatory effects of ANP and RU486 on the complex constitution and downstream EGFR activation. In addition, fewer complexes by RU486 inhibited the activation of EGFR signaling pathway, which was verified by the addition of PD58095 (an inhibitor of MAPK/ERK signaling pathway) (Figure 5c). The decrease in the complex formation further inhibited the PCNA expression, whereas the increased complexes induced by ANP could partly recover the PCNA level (Figure 5d). The results manifested that ANP plays an important role in ovarian functions through NPRA/PGRMC1/EGFR complex formation and activating the downstream MAPK/ERK signaling pathway.

### ANP promoted cell proliferation of human and rat ovarian granulosa cells by activating AP1

**Figure 6:**
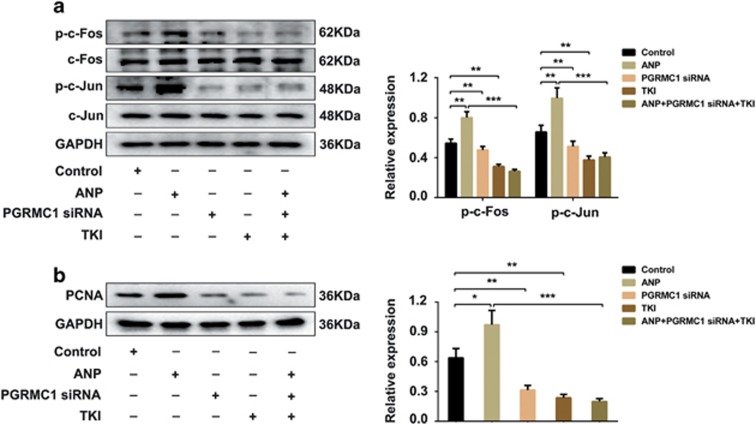
ANP activated AP1 and upregulated the expression of PCNA. KGN cells were treated with ANP, transfected with PGRMC1 siRNA, EGFR inhibitor (TKI), or ANP with PGRMC1 siRNA and TKI, respectively. (**a**) Western blot analysis of activation of AP1 (p-c-Fos, p-c-Jun). (**b**) Level of PCNA was detected by western blot. Significance was indicated by *P<0.05, **P<0.01, ***P<0.001

**Figure 7:**
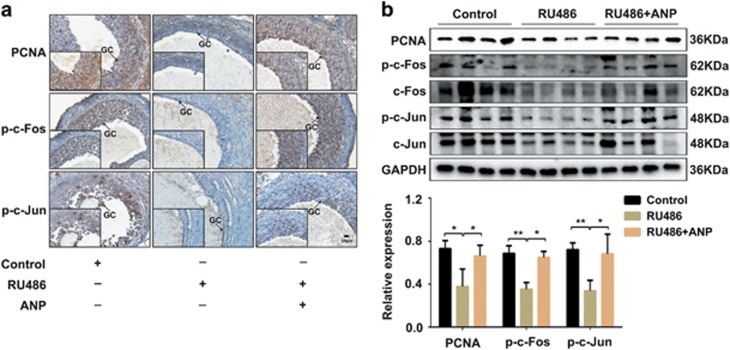
ANP promoted the expression of PCNA by activating AP1 in RU486-induced PCOS rats. PCOS rat model was established by RU486 treatment with or without ANP. (**a**) Representative images of immunohistochemical staining of PCNA and AP1 (p-c-Fos, p-c-Jun) in the ovarian granulosa cells (GC) of PCOS rats and ANP treatment group. Bar=20 *μ*m. (**b**) Western blot analysis of PCNA and activation of AP1 in the ovary tissues of PCOS rats and ANP treatment group. The representative bands and statistical analysis were shown. Significance was indicated by *P<0.05, **P<0.01

Having observed the effects of ANP on the proliferation and apoptosis of KGN cells, we explored whether ANP could promote proliferation through stimulating the activation of transcription factor AP1. KGN cells were pretreated with ANP, PGRMC1 siRNA or TKI (an inhibitor of EGFR activation). The results indicated that ANP increased the activation of AP1 (p-c-Fos and p-c-Jun), and upregulated the expression of PCNA, while PGRMC1 siRNA and TKI inactivated AP1, and downregulated PCNA expression in human KGN cells (Figures 6a and b). The immunohistochemical staining (Figure 7a) and western blot (Figure 7b) results showed decreased levels of PCNA, p-c-Fos and p-c-Jun in the ovary tissues of PCOS rats induced by RU486, whereas the inhibitory effect were reversed in ANP-treated rats. These results suggest that ANP promotes ovarian granulosa cells proliferation by activating AP1 both *in vitro* and *in vivo*.

## Discussion

The relationship between ANP and PCOS has not been well studied. Lauria *et al.*^31^ reported a lower serum ANP level in PCOS patients than in that of age- and BMI-matched controls.^31^ We also found that the serum ANP level decreased in PCOS patients, and in the RU486-induced PCOS rats by ELISA assay. We further confirmed that decreased ANP was correlated with the aberrant morphology and functions by the PCOS rat model. In addition, by *in vivo* and *in vitro* experiments, we found that ANP positively regulated the ovarian functions by promoting proliferation and inhibiting apoptosis of ovarian granulosa cells. The data revealed that ANP maybe a potential therapeutic target for PCOS. Currently, clomiphene citrate (CC) is the first-line treatment for inducing ovulation, and it is a type of anti-estrogen steroid preparation.^38^ However, CC treatment often present many side effects. For example, CC can cause endometrial thinning,^39^ which is not conducive to embryonic implantation, thus leading to decreased pregnancy rates.^40^ ANP is mostly used for heart and kidney diseases by intravenous infusion during surgical operations.^41, 42^ In our study, we found that ANP treatment effectively ameliorated most of the symptoms found in PCOS rats, including polycystic ovaries, hyperandrogenism and hormone production, as well as the pregnancy rate. The study provides the theoretical basis for the treatment of PCOS with ANP, and the pharmacokinetics and clinical trials need to be further conducted.

RU486 is a progesterone receptor antagonist that abolishes the functions of progesterone, such as embryonic development, endometrial receptivity and ovarian functions.^43, 44^ Marions *et al.*^45^ reported that RU486 administration could affect the ovulation process. Furthermore, RU486 could prevent endometrial maturation and proliferation with high doses exceeding 10 mg or repeatedly administered low doses.^46^ Gemzell-Danielsson *et al.*^47^ found that if RU486 was administered at high doses after ovulation, it would inhibit uterine secretory phase progression and embryo implantation through a direct effect on the endometrium. Currently, the regular use of RU486 as contraceptive to avoid pregnancy in young women of childbearing age is common. The long-term use of RU486 may lead to the occurrence of infertility, which can partly explain the increased incidence of infertility in recent years. Thus, we explored the pathogenic mechanism of RU486 in PCOS. A PCOS animal model is useful for detailed molecular studies of changes in histopathology and hormone synthesis, etc. RU486 could induce the similar features of human PCOS, such as anovulation, enlarged ovaries containing atretic follicles or follicular cysts, as well as increased testosterone, estrogen and LH levels.^48^ Lakhani *et al.*^49^ successfully established the PCOS rat model using RU486, which was used to investigate vascular deficits. In our study, we also established the RU486-induced PCOS rat model, which present characteristic polycystic and enlarged ovaries, as well as the consistently changed serum hormones. The inhibitory functions of RU486 on ovary were further confirmed by *in vitro* and *in vivo* study. Furthermore, RU486 impaired endometrial development and pregnancy rate. These results suggest that RU486 may lead to undesirable ovarian development and functions. Therefore, women of reproductive age should use RU486 cautiously.

**Figure 8:**
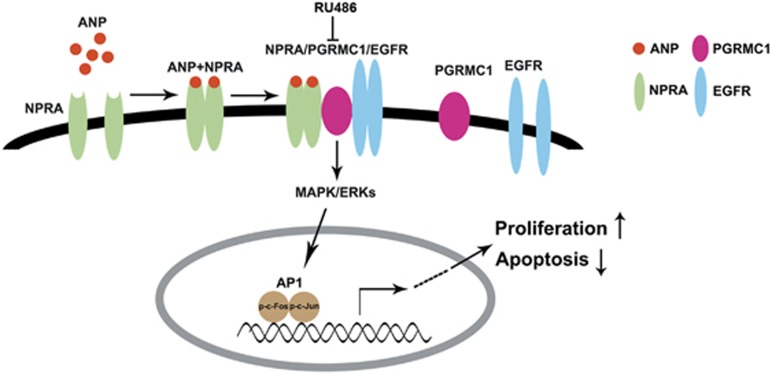
Illustrative model of the mechanism of ANP on proliferation and apoptosis of ovarian granulosa cells

The formation of NPRA/PGRMC1/EGFR complex is the molecular basis by which ANP promotes the growth and inhibits the apoptosis of ovarian granulosa cells. This has not yet been reported. ANP exerts the reproductive activities, like hormone production^50^ and sperm acrosome reaction,^36^ by binding to NPRA/C. PGRMC1 is located on the cell membrane and regulates ovarian development and functions of ovarian granulosa cells. In this study, we found that ANP upregulated NPRA expression and promoted proliferation and inhibited apoptosis, and co-expression of NPRA and PGRMC1 in human ovarian granulosa cells and ovary tissues of PCOS rats were observed. Furthermore, inhibition of PGRMC1 expression by RU486 could be restored by addition of ANP. The above data suggest the co-existence of molecular interaction between NPRA and PGRMC1. EGFR plays a critical role in cell growth and differentiation, and is involved in many reproductive processes, including implantation and decidualization.^51^ Ahmed *et al.*^52^ reported that EGFR was one of the most potent receptor-tyrosine kinases driving tumor proliferation, and PGRMC1 promoted several cancer phenotypes, at least in part, by binding EGFR and stabilizing plasma membrane pools of the receptor. In this study, based on the fact that RU486 inhibited, whereas ANP, NPRA and PGRMC1 promoted the proliferation of ovarian granulosa cells, we further explore whether EGFR is associated with NPRA and PGRMC1-mediated ovary functions. Except the co-localization between NPRA and PGRMC1 in both human ovarian granulosa cells and rat ovary tissues by immunofluorescence observation, the IP results further validated the interaction among NPRA, PGRMC1 and EGFR in KGN cells. RU486 decreased NPRA/PGRMC1/EGFR complex formation. However, ANP could reverse the effects of RU486 on complex formation. The NPRA/PGRMC1/EGFR complex further activated the MAPK/ERK signaling pathway and induced transcription factor AP1 expression and activation, which facilitated proliferation and inhibited apoptosis of KGN cells (Figure 8). These findings demonstrate that ANP improves ovarian functions via the NPRA/PGRMC1/EGFR complex, which is involved in the pathogenesis and treatment strategy of PCOS.

In conclusion, the serum ANP level in PCOS patients was decreased compared with healthy women. ANP treatment reversed the phenotypes of PCOS rats induced by RU486. RU486 inhibited, whereas ANP promoted, the formation of the NPRA/PGRMC1/EGFR complex, which influence the proliferation and apoptosis of ovarian granulosa cells. The study suggests that the use of RU486 may lead to the occurrence of PCOS, and ANP is a novel option as a non-steroid hormonal drug for PCOS treatment.

## Materials and methods

### Ethics statement

The clinical samples obtained from Shengjing Hospital of China Medical University, and signed informed consent forms were obtained from all of the subjects. The research got the approval of the clinical ethics review board of China Medical University and Dalian Medical University. The procedures of animal experiments were carried out in conformity with the guidance for the care and use of laboratory animals in Dalian Medical University.

### Serum sample

Serum samples were obtained from Shengjing Hospital of China Medical University from 2015 to 2016. The diagnosis of PCOS followed the Rotterdam criteria of 2003: oligoovulation, hyperandrogenism or hyperandrogenemia and the morphology of polycystic ovaries. The exclusion of related disorders include Cushing’s syndrome, androgen-producing tumors and congenital adrenal hyperplasia. The healthy control group was with regular menstrual cycle which excluded from other gynecological abnormalities like hirsutism and endocrine dysfunction. The serum samples collected from healthy control group and PCOS patients were used to detect ANP level.

### Cell culture

A steroidogenic human granulosa cell-like tumor cell line (KGN) was obtained from RIKEN (Tokyo, Japan). The KGN cells were maintained in DMEM/F12 supplemented with 10% FBS, 100 U/ml penicillin and 100 *μ*g/ml streptomycin in a humidified atmosphere of 5% CO_2_ at 37 °C. The growth medium was changed every 2–3 days.

### Animal model

Female Sprague-Dawley adult rats (aged 12–14 weeks) were selected, bred and housed with free access to water and food. The rats that showed at least two consecutive estrous cycles by vaginal smear examination were chosen for further study. The rats were injected with RU486 (mifepristone; Sigma-Aldrich, St. Louis, MO, USA) in olive oil (2 mg/0.1 ml/100 g body weight/day) daily subcutaneously, which was begun on the first day of estrous cycle. Control mice were injected with olive oil only. ANP was prepared (2 *μ*g/kg) and applied by intraperitoneal administration. Samples, including serum and ovarian tissues, were collected for further study.

### Transfection

Synthetic siRNA of PGRMC1 (GenePharma, Shanghai, China) was delivered into cells with Lipofectamine 2000 (Invitrogen, Carlsbad, CA, USA) according to the protocols. The siRNA sequences of PGRMC1 were 5′-GGUGUUCGAUGUGACCAAATT-3′ and 5′-UUUGGUCACAUCGAACACCTT-3′. The protein and RNA samples were collected and experiments were performed 48 h after transfection.

### Immunofluorescence

Frozen tissue sections from control and PCOS rat model were fixed in 4% paraformaldehyde at room temperature following with 20% sucrose for 48 h. The tissues were embedded with OCT and sliced at −20 °C. After fixed with acetone, goat serum was used to block at room temperature for 2 h. The primary antibodies mouse anti-NPRA (1:50; Santa Cruz Biotechnology, CA, USA) and NPRC (1:100; Santa Cruz Biotechnology, CA, USA), rabbit anti-PGRMC1 (1:100; Cell Signaling, Danvers, MA, USA) and PGRMC2 (1:50; Cell Signaling) were incubated at 37 °C for 2 h. Washing with PBS, TRITC-conjugated goat anti-rabbit IgG (1:100; ZSGB-BIO, Shanghai, China) and FITC-conjugated goat anti-mouse IgG (1:100; Sigma-Aldrich, St. Louis, MO, USA) were incubated for 1 h at 37 °C following treatment with DAPI (1:1000; Beyotime, Shanghai, China) for 3 min. The immunofluorescent images were taken with an inverted microscope (Olympus, Tokyo, Japan).

Cells grown on coverslips were fixed with 4% paraformaldehyde for 20 min at room temperature following incubation with 0.1% Triton X-100 for 15 min. After blocking with goat serum, cells were incubated with rabbit anti-PCNA (1:200; Proteintech, Wuhan, China) at 4 °C overnight. TRITC-conjugated goat anti-rabbit IgG was incubated for 1 h and DAPI (1:1000) for 3 min. Immunofluorescent staining was photographed with an inverted microscope.

### ELISA

Commercial ELISA kits (Elabscience, Wuhan, China) were used to detect the hormones (progesterone, testosterone, estrogen, LH, FSH) and ANP levels both in human serum and rat serum according to the manufacturer’s instructions. The absorbance at 450 nm was measured and recorded. Three samples from each group were tested.

### RNA isolation and real-time PCR

Total RNA was isolated using TRizol reagent (TaKaRa, Dalian, China) according to the manufacture’s manual. A total of 1 *μ*g RNA was used for cDNA synthesis with PrimeScript RT Master Mix Perfect Real Time Kit (TaKaRa). The primers sequences of human were as follows: NPRA, forward 5′-GGGATACAGTCAACACAGCCTCAA-3′, reverse 5′-CGAAGCTCCAGCTCGAAAC-3′ NPRC, forward 5′-AGATGCCAACGGAGACCGATA-3′, reverse 5′-ACATTCGGCCGCATTTCA-3′ PGRMC1, forward 5′-CGGGCTGCTGCATGAGATT-3′, reverse 5′-GCACGATCTTGTAGAGCAGGAA-3′ PGRMC2, forward 5′-GGCAATGTTATTTAACAGGTCACCA-3′, reverse 5′-TCCACAACCAGTCTTCAGCAA-3′. The primers sequences of rats were as follows: NPRA, forward 5′-TGGAGACACAGTCAACACAGCTTC-3′, reverse 5′-TCCAGCACAGCCTTGGTCTC-3′ NPRC, forward 5′-ACACGGCATGACCAGTGGAG-3′, reverse 5′-TCAGCAGGGTGACTGTTTGGAG-3′ PGRMC1, forward 5′-CACCTGGTAATTGGCAGTTGGA-3′, reverse 5′-ACCACATAACCATTGCCCTGCTA-3′ PGRMC2, forward 5′-TTTGAACGCAGTGCAGATGG-3′, reverse 5′-GGTACGAGTTGCAGTTCTGAAGG-3′.

### Western blot

Protein samples were extracted with lysis buffer. 15 *μ*g protein samples from each group were loaded onto the SDS polyacrylamide gel for electrophoresis and transferred to NC membranes (Millipore, Billerica, MA, USA). After blocking in 5% non-fat milk at room temperature for 2 h, primary antibodies: NPRA (1:1000; Santa Cruz Biotechnology), NPRC (1:1000; Santa Cruz Biotechnology), PGRMC1 (1:1000; Cell Signaling), PGRMC2 (1:1000), Bax (1:2000; Proteintech), Bcl-2 (1:2000; Proteintech), Caspase 8 (1:1000; Proteintech), Caspase 9 (1:1000; Proteintech), PCNA (1:2000; Proteintech), EGFR (1:500; Proteintech), p-EGFR (1:500; Cell Signaling), ERK 1/2 (1:500; Beyotime Biotechnology), p-ERK 1/2 (1:500; Beyotime Biotechnology), p-c-Fos (1:500; Cell Signaling), c-Fos (1:500; Cell Signaling), p-c-Jun (1:500; Cell Signaling), c-Jun (1:500; Cell Signaling) and GAPDH (1:2000; Proteintech) were incubated at 4 °C overnight. Next day, secondary antibodies (1:2000; Cell Signaling) were incubated for 45 min at room temperature. ECL detection system was used to visualize the bands. All experiments were repeated at least three separate times.

### Immunohistochemistry

Paraffin-embedded rat ovary tissues were prepared, followed by deparaffinization and rehydration. After antigen exposed, tissue slides were incubated with 3% H_2_O_2_ for 10 min and goat serum for 30 min to block non-specific binding. Primary antibodies: ANP (1:100; Abcam, Cambridge, UK), NPRA (1:100; Santa Cruz Biotechnology), NPRC (1:100; Santa Cruz Biotechnology), PGRMC1 (1:50; Cell Signaling), PGRMC2 (1:50; Cell Signaling), PCNA (1:200; Proteintech), p-c-Fos (1:50; Cell Signaling), c-Fos (1:50; Cell Signaling), p-c-Jun (1:50; Cell Signaling) and c-Jun (1:50; Cell Signaling) were incubated overnight at 4 °C. The second antibody was incubated for 40 min after washing with PBS. The signals were visualized with DAB wherein yellowish-brown stain indicated as a positive result. The negative control was obtained by replacing primary antibody with PBS. Images were taken under an inverted microscope.

### Cell Counting Kit-8 assay

1 × 10^3^ cells were plated in a 96-well plate and treated with RU486 (10^−5^ M), ANP (10^−7^ M) or PGRMC1 siRNA (50 nM). Cell Counting Kit-8 (CCK-8, Dojindo Laboratories, Kumamoto, Japan) was added to the cell culture medium. Absorbance at 450 nm was measured and data were recorded after incubation at 37 °C for 2 h. All tests were repeated at least three times.

### Colony formation assay

Cells were plated in six-well plates containing DMEM/F12 with 10% FBS after treatment with RU486 (10^−5^ M), ANP (10^−7^ M) or PGRMC1 siRNA (50 nM). Colonies were fixed and stained with crystal violet for 20 min after growing for 7–10 days. Images of surviving colonies were captured and colony numbers were counted.

### TUNEL assay

Apoptotic cells analysis was performed using the terminal deoxynucleotidyl transferase-mediated dUTP nick-end labeling (TUNEL) staining kit (Roche, Mannheim, Germany) according to the protocol. Cells grown on glass coverslips were fixed with 4% paraformaldehyde followed by primary antibody incubation. DAPI was used to counterstain the nuclei. The TUNEL-positive cells had a pyknotic nucleus with dark green fluorescent staining, which was an indicator of apoptosis. Images were taken with an inverted microscope.

### Immunoprecipitation

Cells were lysed with RIPA buffer (Beyotime) containing protease and phosphatase inhibitors. Cell lysates were incubated with NPRA (2 *μ*g/ml; Santa Cruz Biotechnology) or PGRMC1 (2 *μ*g/ml; Cell Signaling) antibodies. The IPs were performed overnight, followed with protein A/G sepharose added (Thermo Fisher Scientific, Waltham, MA, USA). The beads were collected by centrifugation and then washed with lysis buffer. Samples were boiled in SDS-PAGE buffer for 5 min to elute the protein bound to the beads. The final immunoprecipitaions were subjected to western blot analysis.

### Fertility assessment

To assess the effects of ANP on fertility, female rats from RU486-induced PCOS rats and ANP treatment group mated with male rats. Successful mating was defined by the discovery of a vaginal plug next day. Female rats were killed to confirm pregnancy at gestational day 10 (GD10).

### Scanning electron microscope

Endometrium tissues collected from PCOS rats and ANP-treated rats at gestational day 4 (GD4) were fixed in 4% paraformaldehyde and postfixed in 2% osmium tetroxide. Then dehydrated with a series of ethanol: 95% ethanol, absolute ethanol and acetone. The surface of the endometrium was scanned with a scanning electron microscope (S-3700N; Hitachi, Tokyo, Japan) after coated with gold.

### Statistical analysis

At least three independent experiments were performed for each group. Results were expressed as mean±S.E.M. from at least three independent experiments. Differences between groups were analyzed with Student’s *t*-test. The significance was defined as *P*<0.05. SPSS 22.0 (IBM, Chicago, IL, USA) was used for the analysis.

## Publisher’s Note

Springer Nature remains neutral with regard to jurisdictional claims in published maps and institutional affiliations.

## Figures and Tables

**Table 1:** Endocrine parameters in serum of RU486-induced PCOS and ANP treatment rats

	**P (ng/ml)**	**T (ng/ml)**	**E**_**2**_ **(pg/ml)**	**FSH (ng/ml)**	**LH (mIU/ml)**
Control group	438.40±109.42	10.654±1.17	634.63±70.49	210.00±20.55	135.37±26.94
RU486 group	204.78±44.09	16.274±1.25	1247.00±236.80	242.32±28.65	296.40±47.95
ANP group	378.41±13.60	12.48±1.61	895.88±151.45	239.28±34.21	85.07±24.59
*P* (RU486/Control)	<0.01	<0.001	<0.01	>0.05	<0.001
*P* (ANP/RU486)	<0.001	<0.01	<0.05	>0.05	<0.001

*P*: statistical significance (*P<0.05; **P<0.01; ***P<0.001)
